# Use of menopausal hormone therapy before and after diagnosis and ovarian cancer survival—A prospective cohort study in Australia

**DOI:** 10.1002/ijc.35154

**Published:** 2024-09-02

**Authors:** Renhua Na, Susan J. Jordan, Anna DeFazio, Merran Williams, Karen Livingstone, Andreas Obermair, Michael Friedlander, Peter Grant, Penelope M. Webb, for the OPAL Study Group

**Affiliations:** ^1^ Population Health Program QIMR Berghofer Medical Research Institute Brisbane Australia; ^2^ School of Public Health University of Queensland Brisbane Australia; ^3^ Department of Gynaecological Oncology Westmead Hospital Westmead Australia; ^4^ Centre for Cancer Research The Westmead Institute for Medical Research Westmead Australia; ^5^ The Daffodil Centre The University of Sydney, A Joint Venture with Cancer Council NSW Sydney Australia; ^6^ Consumer Representative Brisbane Australia; ^7^ Consumer Representative Melbourne Australia; ^8^ Queensland Centre for Gynaecological Cancers Royal Brisbane and Women's Hospital Brisbane Australia; ^9^ Department of Medical Oncology Prince of Wales Hospital and Prince of Wales Clinical School UNSW Sydney Sydney Australia; ^10^ Gynaecological Oncology Unit Mercy Hospital for Women Melbourne Australia

**Keywords:** menopausal hormone therapy, ovarian cancer, propensity score, quality of life, survival

## Abstract

Menopausal hormone therapy (MHT) use before ovarian cancer diagnosis has been associated with improved survival but whether the association varies by type and duration of use is inconclusive; data on MHT use after treatment, particularly the effect on health‐related quality of life (HRQOL), are scarce. We investigated survival in women with ovarian cancer according to MHT use before and after diagnosis, and post‐treatment MHT use and its association with HRQOL in a prospective nationwide cohort in Australia. We used Cox proportional hazards regression to estimate hazard ratios (HR) and 95% confidence intervals (CI) and propensity scores to reduce confounding by indication. Among 690 women who were peri‐/postmenopausal at diagnosis, pre‐diagnosis MHT use was associated with a significant 26% improvement in ovarian cancer‐specific survival; with a slightly stronger association for high‐grade serous carcinoma (HGSC, HR = 0.69, 95%CI 0.54–0.87). The associations did not differ by recency or duration of use. Among women with HGSC who were pre‐/perimenopausal or aged ≤55 years at diagnosis (*n* = 259), MHT use after treatment was not associated with a difference in survival (HR = 1.04, 95%CI 0.48–2.22). Compared to non‐users, women who started MHT after treatment reported poorer overall HRQOL before starting MHT and this difference was still seen 1–3 months after starting MHT. In conclusion, pre‐diagnosis MHT use was associated with improved survival, particularly in HGSC. Among women ≤55 years, use of MHT following treatment was not associated with poorer survival for HGSC. Further large‐scale studies are needed to understand menopause‐specific HRQOL issues in ovarian cancer.

## Graphical abstract

What's new?

There are limited data about the effects and safety of menopausal hormone therapy for women with ovarian cancer. This nationwide prospective cohort study shows that pre‐diagnosis menopausal hormone therapy use is associated with improved survival in peri‐/postmenopausal women with ovarian cancer, particularly high‐grade serous carcinoma. Moreover, among women aged less than 55 years at diagnosis, menopausal hormone therapy use after treatment is not associated with poorer survival in women with high‐grade serous carcinoma, suggesting that menopausal hormone therapy could be considered to control menopausal symptoms in this group.

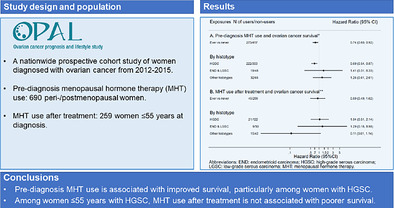

## 1 INTRODUCTION

Women with ovarian cancer are often diagnosed with advanced stage disease, and their overall 5‐year survival is less than 50%.^1^, ^2^ Treatment usually requires a combination of surgery, including oophorectomy, and chemotherapy and, as a result, women who are premenopausal before diagnosis experience immediate menopause due to an acute and profound reduction in endogenous oestrogen.

Management of menopausal symptoms in women with ovarian cancer is challenging because of uncertainty regarding the potential harms and benefits of systemic menopausal hormone therapy (MHT) after diagnosis. One concern is that MHT use appears to increase the risk of developing ovarian cancer, particularly the serous and endometrioid histotypes.^3^, ^4^, ^5^ However, pre‐diagnosis MHT use has also been associated with improved ovarian cancer survival^6^, ^7^, ^8^, ^9^ especially with more than 5 years of use,^10^, ^11^, ^12^, ^13^ with one study reporting that MHT users were less likely to have residual disease following primary surgery, a significant prognostic factor.^12^ There is currently little information on MHT use after diagnosis, particularly among those who are pre‐menopausal at diagnosis.

Previous studies including two small clinical trials have indicated a potential survival benefit with post‐diagnosis MHT use.^9^, ^14^, ^15^, ^16^, ^17^, ^18^, ^19^ However, most of these studies did not account for pre‐diagnosis MHT use which, as noted above, is associated with improved survival; and some^9^, ^18^ included postmenopausal women who are less likely to require MHT to manage menopausal symptoms. Furthermore, most studies have not considered whether any survival benefit may vary by histotype. Anti‐oestrogen and endocrine therapies appear to have some benefit for women with low‐grade serous carcinomas,^20^ indicating that MHT might be contraindicated for this group.

Among women who develop significant menopausal symptoms after treatment, appropriate use of oestrogen should provide significant relief and therefore improve health‐related quality of life (HRQOL) but data evaluating this are scarce. Only one small non‐blinded trial (*n* = 75) has reported the relation between MHT use and HRQOL after ovarian cancer treatment, suggesting better HRQOL among MHT users.^21^

Using data from a nationwide prospective cohort study of women with ovarian cancer, the primary aims of this study were to investigate the association between MHT use *before* diagnosis and ovarian cancer specific survival, and whether use *after* diagnosis was associated with ovarian cancer specific survival or HRQOL.

## 2 METHODS

### 2.1 Study population

The Ovarian cancer Prognosis And Lifestyle (OPAL) study has been described previously.^22^ Briefly, the OPAL study is a national prospective cohort study of Australian women (aged 18–79 years) newly diagnosed with invasive epithelial ovarian, primary peritoneal or fallopian tube cancer from 2012 to 2015. After excluding 219 (15%) women who were too unwell to participate, unable to complete the study documents in English, or unable to provide informed consent, 958 women (78% of those approached) were eligible and consented to participate.

### 2.2 Data collection

Women completed a baseline questionnaire at recruitment. This captured demographic information (e.g., age and education), data on lifestyle factors including smoking status one‐year before diagnosis, height, average weight in the 5 years before diagnosis, prior cancer history, comorbidities (e.g. diabetes, hypertension, cardiovascular diseases) and medication use. Women were asked if they had ever used MHT before diagnosis, the types of MHT (oestrogen‐only (ET), oestrogen plus progestin or progestogen‐only therapy (E‐P/P)) and duration of use.

During follow‐up, women completed questionnaires every 3 months for the first 12 months and annually thereafter until 48 months. The questionnaires assessed regular use of MHT and the type of MHT taken in the previous 4 weeks as well as HRQOL. Records were also electronically linked to the Pharmaceutical Benefits Scheme (PBS) for women who provided specific consent. Linkage provided data on all medicines listed on the PBS Schedule dispensed after 1 July 2012. Services Australia supplied the linked PBS data.

Although postmenopausal symptoms were not specifically addressed in the follow‐up questionnaires, women completed a number of instruments, which include questions related to postmenopausal symptoms. The Functional Assessment of Cancer Therapy—general instrument (FACT‐G) includes 27 items providing a total QOL score (0–108) and subscales for physical (PWB, 0–28), functional (FWB, 0–28), social/family (SWB, 0–28) and emotional well‐being (EWB, 0–24) with higher scores indicating better QOL. It also assesses satisfaction with sex life (GS7) which was categorised as ‘not satisfied’, ‘somewhat or quite a bit’ and ‘very much satisfied’. Fatigue was evaluated using the Functional Assessment of Chronic Illness Therapy (FACIT)—fatigue scale (0–52) with higher scores indicating better function or less fatigue. Finally, insomnia severity was measured using the Insomnia Severity Index (ISI), a 7‐item self‐report screening tool with scores categorised as no significant insomnia (0–7), subthreshold insomnia (8–14), moderate (15–21) and severe insomnia (22–28).^23^ The FACT‐G, FACIT‐fatigue and ISI questionnaires have demonstrated strong validity and reliability and are widely used for measuring HRQOL.^24^, ^25^, ^26^

Data on histotype, the International Federation of Gynaecology and Obstetrics (FIGO) stage, grade, residual disease after primary cytoreduction, chemotherapy treatment, vital status, cause and date of death were collected through annual review of medical records (to December 2020).

### 2.3 Assessment of exposure

For analyses of pre‐diagnosis MHT use, we assessed ever or never use, recency, duration of use and types of MHT (exclusive ET use, E‐P/P and unknown type). Women were classified as recent users if they self‐reported using any systemic MHT within the 2 years before diagnosis and as former users if they had stopped more than 2 years before diagnosis.

MHT use in the first year after diagnosis (ever/never) was ascertained through follow‐up questionnaires and PBS data. We primarily used self‐reported data but also considered PBS prescriptions for oestrogen (Anatomical Therapeutical Chemical classification system code G03CA; only one woman had a prescription for a progestogen) where questionnaire data were incomplete because women had withdrawn (4% of follow‐up time points) or missing (10% of follow‐up time points). PBS data were available for 199 women (75% of those in the post‐diagnosis analysis) and 50% of the missing time points. Among those with questionnaire and PBS data, there was substantial agreement between self‐reported MHT use and information from the PBS (kappa = 0.72, 95% CI 0.59–0.84). Women were classified as MHT users if they either self‐reported using MHT or had at least two prescriptions for MHT dispensed within 90 days^27^ prior to the date they completed the questionnaire (or the date the questionnaire was due if it was missing).

### 2.4 Follow‐up and outcome of interests

The primary outcome of interest was ovarian cancer specific survival (OVS), defined as the time from the start of follow‐up until the date of the last follow‐up or death, with deaths from any other cause being censored. We also considered overall survival (OS) including deaths from any cause and progression‐free survival (PFS), defined as the time from the start of follow‐up until the first confirmed progression or recurrence of disease. For pre‐diagnosis analyses, follow‐up time accrued from date of diagnosis and was left‐truncated to the date the baseline questionnaire was completed. For post‐diagnosis analyses, follow‐up started from 12 months after diagnosis and was left‐truncated to the date the 12‐month questionnaire was completed. Follow‐up ended at the date of death or the date when women were last known to be alive. The secondary outcome of interest was HRQOL.

### 2.5 Statistical analysis

**FIGURE 1:**
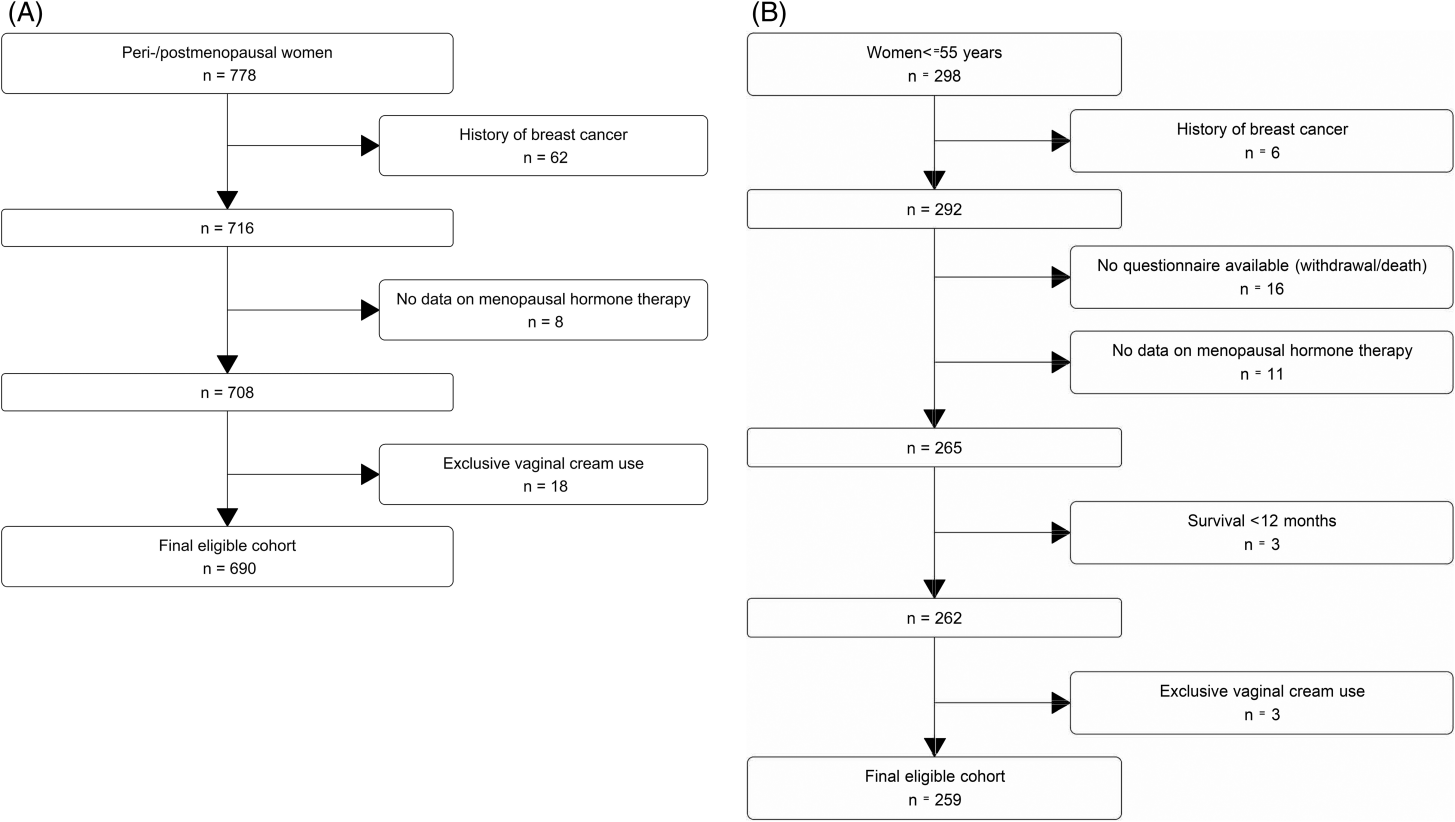
Flow diagram of the study cohort for use of menopausal hormone therapy and ovarian cancer survival (A) pre‐diagnosis analysis; (B) post‐diagnosis analysis.

We restricted the analyses of pre‐diagnosis MHT use to women who self‐reported that they were peri‐/postmenopausal at diagnosis or were over 55 years at diagnosis (*n* = 778). To assess post‐diagnosis MHT use, we restricted the analysis to women who were ≤ 55 years at diagnosis, treated with surgery and alive at 12 months after diagnosis (*n* = 298). We were unable to assess post‐diagnosis MHT use among women over 55 years at diagnosis, as only five women (1%) in this group used systemic MHT during the first 12 months after diagnosis. For all analyses we excluded women with a history of breast cancer (*n* = 62 for pre‐diagnosis use and 6 for post‐diagnosis use); those missing data on MHT use (*n* = 8 and 26) and women who exclusively used vaginally‐administered ET (*n* = 18 and 3). This is because vaginally‐administered ET relieves local symptoms without increasing serum oestradiol levels^28^ (except for high‐dose vaginal cream which was not available/reported during the period of our study), and so is unlikely to be associated with survival. Although women who exclusively used vaginally‐administered ET might be considered to be non‐users of systemic MHT, they differed from non‐users in a number of ways and so were not included in the non‐user group; for example, vaginal ET users were older than MHT non‐users (median age 66 vs. 63 years). The number of exclusive vaginal ET users was too small to analyse this group separately. The flow diagrams showing exclusions for pre‐ and post‐diagnosis analyses are shown in Figure 1A,B.

Finally, for the HRQOL analysis, we restricted the analysis to women who completed at least two questionnaires post‐treatment; for MHT users this had to include one questionnaire prior to initiating MHT (T‐0) and one questionnaire 1–3 months after they initiated MHT post‐treatment (T‐1). Characteristics of women with and without MHT use were compared using Student's *t*‐tests, Wilcoxon rank‐sum tests, Pearson chi‐square tests or Fisher exact tests as appropriate.

We used multivariable Cox proportional hazards regression models to estimate adjusted hazard ratios (HRs) and 95% confidence intervals (CIs) for survival outcomes. For analyses of pre‐diagnosis MHT use, we adjusted for age at diagnosis (<55, 55–59, 60–64, 65–69, 70+), body mass index (BMI, <25, 25–29.9, 30+ kg/m^2^), smoking (never, former, current) and hysterectomy status before diagnosis (yes, no, unknown) as this influences the type of MHT used. We stratified by FIGO stage at diagnosis (I & II, III & IV) as the baseline hazard varies greatly by stage. The type of MHT was unknown for 104 women (15%), including 6 women also missing duration of use, and one missing data on the recency of use. An additional 6 women were missing other variables: MHT duration (*n* = 2), residual disease (*n* = 3), and hysterectomy status (*n* = 1). Supplementary Table 1 compares the demographic and clinical characteristics of women with and without missing data. Women with missing data were older and, as a result, more likely to have hypertension, advanced disease and HGSC. However, they were less likely to be obese or current smokers compared to those with complete data. Additionally, those missing data were more likely to have a history of hysterectomy, which affects type of MHT used, before diagnosis. Among MHT users, where most missing data occurred, women with missing data were older at diagnosis, had more comorbidities such as hypertension, and were less likely to be current smokers (data not shown). We imputed missing data for these variables using the multiple imputation with chained equations (MICE) package in R (Supplementary Methods). We also calculated the difference in the adjusted restricted mean survival time (RMST)^29^ at 5 years between pre‐diagnosis MHT users and non‐users, both overall and for the HGSC group specifically.

As it has been suggested that residual disease might be a causal mediator between pre‐diagnosis MHT use and survival,^12^ we conducted a mediation analysis based on the potential outcomes framework proposed by Lange et al.,^30^ restricting to women with advanced HGSC. Three women missing data on residual disease and hysterectomy were excluded from this analysis. This approach separates the total effect of a given exposure into a natural direct and indirect effect through mediators. We used bootstrapping to calculate 95% CIs using 2000 replications.^31^

For analyses of post‐diagnosis MHT use, we included the same confounders (except hysterectomy), and also adjusted for FIGO Stage (I & II, III & IV), residual disease (none, any) and pre‐diagnosis MHT use (ever, never). Nine women (3%) with missing data on residual disease were excluded. Further adjustment for any other variables including treatment for ovarian cancer (surgery only, neoadjuvant chemotherapy, adjuvant chemotherapy) did not alter the results. We first used a time‐varying approach, allowing women to transition between MHT ‘non‐use’ and ‘use’ over time incorporating a 12‐month lag for exposed person‐time. This approach allowed us to account for changes in MHT use over time and minimise the potential for reverse causation.^32^, ^33^, ^34^ Specifically, women were classified as users or non‐users at 12 months based on their exposure at 3–9 months post‐diagnosis, their exposure status was then updated annually based on their use at the previous time‐point (e.g., use at 24 months was determined based on reported use and/or prescriptions around 12 months). We also considered MHT use as a fixed binary variable (yes, no) based on use during the first 12 months. We then evaluated change of MHT use from before diagnosis to 12 months after diagnosis and classified women as never users, those who only used MHT before diagnosis, those who only used it after diagnosis (new users) and continuous users.

We conducted stratified analyses investigating potential effect modification by stage and histotype.^35^ We tested the assumption of proportional hazards in the Cox proportional hazard regression models based on the scaled Schoenfeld residuals, and there was no evidence of violation of this assumption.

### 2.6 Sensitivity analysis

We repeated the survival analyses using a propensity score approach to reduce the effects of confounding by indication^35^ (Supplementary Methods). For post‐diagnosis MHT use, we restricted the analysis to women with a complete or partial response to primary treatment (based on normalisation of cancer antigen 125^36^ and/or no residual tumour) because women with progressive disease might be less likely to start MHT use. We also excluded 33 women who used endocrine therapy (e.g. tamoxifen and letrozole) and three women with history of coronary heart disease (CHD) as these are contraindications to MHT use.

Statistical analyses were performed in SAS version 9.4 (SAS Institute, Cary, North Carolina) and R statistical software (version 4.0.2, R Development Core Team, 2020).

## 3 RESULTS

The study population for the pre‐diagnosis analysis included 690 women who were peri‐/postmenopausal at diagnosis and eligible for inclusion in the analysis (Figure 1A). During a median follow‐up time of 5 years (interquartile range 3–6), a total of 395 (57%) women died, with most (381, 96% of all deaths) dying from ovarian cancer. Table 1 compares the demographic and clinical characteristics of never (60%) and ever MHT users (40%). MHT users were significantly older, more likely to have advanced disease and HGSC but less likely to be obese. As expected, MHT users were more likely to have a history of hysterectomy before diagnosis than never users (34% vs. 16%).

**TABLE 1:** Baseline characteristics of peri‐/postmenopausal women with ovarian cancer, by MHT use before diagnosis.

Variable^a^	Total (*n* = 690)	Never use (*n* = 417)	Ever use (*n* = 273)
Age at diagnosis, years (Mean, SD)	63 (8)	62 (8)	66 (7)

	*N* (%)		*N* (%)		*N* (%)	
Menopause status						
Peri‐menopause^b^	44 (6)		41 (10)		3 (1)	
Post‐menopause	646 (94)		376 (90)		270 (99)	
FIGO Stage						
I & II	168 (24)		118 (28)		50 (18)	
III & IV	522 (76)		299 (72)		223 (82)	
Histotype						
High‐grade serous carcinoma	499 (77)		280 (74)		219 (81)	
Non high‐grade serous carcinoma	147 (23)		96 (26)		51 (19)	
Residual disease						
None	380 (55)		230 (57)		150 (57)	
Any	285 (45)		172 (43)		113 (43)	
Unknown	25		15		10	
BMI 5 years before diagnosis (kg/m^2^)						
<25	287 (42)		165 (40%)		122 (45%)	
25–29.9	234 (34)		135 (32%)		99 (36%)	
30+	169 (24)		117 (28%)		52 (19%)	
Smoking status 1 year before diagnosis						
Never	363 (53)		219 (53)		144 (53)	
Former	247 (36)		140 (34)		107 (39)	
Current	80 (11)		58 (14)		22 (8)	
Highest level of education						
High school	349 (51)		206 (50)		143 (53)	
Technical college	171 (25)		101 (24)		70 (26)	
University	167 (24)		107 (26)		60 (22)	
Age at menarche, years (Mean, SD)	13 (4)		13 (5)		13 (2)	
Hysterectomy prior to diagnosis						
No	531 (77)		351 (84)		180 (66)	
Yes	158 (23)		65 (16)		93 (34)	
Charlson comorbidity score						
0	505 (73)		306 (73)		199 (73)	
1	119 (17)		67 (16)		52 (19)	
2+	66 (10)		44 (11)		22 (8)	
Medical history						
Coronary heart disease	22 (3)		15 (4)		7 (3)	
Hypertension	266 (39)		149 (36)		117 (43)	
Diabetes	52 (8)		34 (8)		18 (7)	
Family history of breast or ovarian cancer						
No	559 (81)		334 (81)		225 (82)	
Yes	127 (19)		79 (19)		48 (18)	

Abbreviations: BMI, body mass index; SD, standard deviation.

^a^ Continuous variables presented as mean (standard deviation) and categorical variables presented as *n* (%).

^b^ Including three women who self‐reported as pre‐menopause but were over 55 years at diagnosis.

Table 2 shows the associations between pre‐diagnosis MHT use and OVS. Ever MHT use was associated with a 26% improvement in OVS (HR 0.74, 95% CI 0.60–0.92) compared to never use. Although the survival benefit associated with E‐P/P (HR = 0.67) appeared to be stronger than for E‐only (HR = 0.84), the difference was not statistically significant. The HRs did not differ appreciably by MHT recency or duration of use and were also similar for OS (data not shown) and PFS (Supplementary Table 3). Results did not change materially after multiple imputation for MHT type and recency/duration (Supplementary Table 2). We observed slight attenuation when using propensity scores to reduce potential confounding by indication and histotype (ever MHT use and OVS: HR 0.79, 95% CI 0.62–0.98). Although the association appeared somewhat stronger among women with early‐stage (HR 0.46, 95% CI 0.16–1.33) than advanced disease (HR 0.75, 95% CI 0.60–0.93), the CI for the estimate for early stage disease was very wide (Supplementary Table 4). Results restricted to HGSC were similar (ever use: HR 0.69, 95% CI 0.54–0.87) (Table 2); in contrast, among women with non‐HGSC (*n* = 165), MHT use was associated with a non‐significant increase in ovarian cancer mortality (HR 1.34, 95% CI 0.71–2.54). Specifically, among the small group of women with endometrioid and low‐grade serous carcinomas (*n* = 67), the HR for ever MHT use was estimated at 1.41 (95% CI 0.31–6.33). For those with mucinous and clear‐cell carcinomas (*n* = 57), the HR was 1.76 (95% CI 0.41–7.54). In the mediation analysis restricted to women with advanced HGSC (*n* = 457), MHT use was associated with 31% better OVS; only 11% of this association was attributed to indirect pathways mediated by residual disease (Supplementary Table 5). Overall, the difference in 5‐year RMST between for MHT users and non‐users was 1.5 months (95% CI –1.4–4.4). However, among women with HGSC, the difference was 3.7 months (95% CI 0.5–7.0, *p* = 0.03) in favour of MHT users (45.8 vs. 42.1 months, respectively).

**TABLE 2:** MHT use prior to diagnosis and ovarian cancer‐specific survival among peri‐/postmenopausal women (*n* = 690).

			All (*n* = 690)		HGSC (*n* = 525)		Non‐HGSC (*n* = 165)	
Variable^a^	Total^b^ (*n*, %)	Deaths (*n*, %)	HR	95%CI	HR	95%CI	HR	95%CI
MHT use								
Never	417 (60)	224 (62)	1.0		1.0		1.0	
Ever	273 (40)	137 (38)	0.74	0.60–0.92	0.69	0.54–0.87	1.34	0.71–2.54
Recency of use								
Former	178 (26)	94 (26)	0.74	0.57–0.95	0.67	0.51–0.88	1.40	0.69–2.84
Current/recent	94 (14)	43 (12)	0.76	0.55–1.04	0.73	0.52–1.02	1.23	0.47–3.23
MHT type								
ET	87 (15)	39 (13)	0.84	0.59–1.21	0.90	0.61–1.33	1.01	0.37–2.75
E‐P/P	82 (14)	41 (14)	0.67	0.48–0.93	0.62	0.44–0.88	1.64	0.56–4.78
MHT duration^c^								
<5 years	128 (19)	60 (17)	0.70	0.53–0.93	0.63	0.46–0.86	1.30	0.60–2.78
5+ years	137 (20)	72 (20)	0.77	0.59–1.01	0.72	0.55–0.96	1.35	0.55–3.35

Abbreviations: CI, confidence interval; E‐P/P, oestrogen plus progestin or progestogen‐only therapy; ET, oestrogen‐alone hormone therapy; HGSC, high‐grade serous carcinoma; HR, hazard ratio; MHT, menopausal hormone therapy.

^a^ All models adjusted for age (<55, 55–59, 60–64, 65–69, 70+), body mass index (<25, 25–29, 30+ kg/m^2^) smoking (never, former, current), history of hysterectomy prior to diagnosis (yes, no) and stratified by FIGO Stage (I & II, III & IV). Further adjustment for any other variables did not make any difference.

^b^ The counts in sub‐categories may not add up to the total number due to missing data.

^c^ Mean (standard deviation) duration of MHT: <5 years = 20 months (15.3); 5+ years = 12 years (6.9).

Of the 259 women included in the analysis of MHT use after diagnosis (Figure 1B), 101 (39%) women died including 99 (98% of all deaths) from ovarian cancer. Table 3 shows the demographic and clinical characteristics of the women by MHT use during the first 12 months after diagnosis. Most women (79%) never used MHT, 4% used MHT before diagnosis but discontinued after diagnosis, 3% continued using MHT and 14% initiated MHT after diagnosis. MHT users were younger, more highly educated and less likely to have residual disease (Table 3).

**TABLE 3:** Characteristics of women age ≤ 55 years at diagnosis by use of MHT after diagnosis (*n* = 259).

Variable^a^	Total (*n* = 259)	No use (*n* = 214)	Any use (*n* = 45)
Age at diagnosis, years (Mean, SD)	49 (44–52)	48 (6)	44 (8)

	*N* (%)	*N* (%)	*N* (%)	
FIGO Stage				
I & II	115 (44)	91 (43)	24 (53)	
III & IV	144 (56)	123 (57)	21 (47)	
Histotype				
High‐grade serous carcinoma	143 (55)	122 (57)	21 (47)	
Non high‐grade serous carcinoma	116 (45)	92 (43)	24 (53)	
Residual disease				
None	183 (71)	145 (68)	38 (84)	
Any	74 (29)	67 (32)	7 (16)	
Response to primary treatment				
Complete or partial response	245 (95)	204 (95)	41 (91)	
Stable disease or progression	14 (5)	10 (5)	4 (9)	
Primary treatment				
Surgery only, no chemotherapy	31 (12)	27 (13)	4 (9)	
Neoadjuvant therapy	50 (19)	46 (21)	4 (9)	
Adjuvant chemotherapy	178 (69)	141 (66)	37 (82)	
BMI 5 years before diagnosis (kg/m^2^)				
<25	130 (50)	102 (48)	28 (62)	
25–29.9	61 (24)	51 (24)	10 (22)	
30+	68 (26)	61 (29)	7 (16)	
Smoking status 1 year before diagnosis				
Never	142 (55)	122 (57)	20 (44)	
Former	75 (29)	61 (29)	14 (31)	
Current	42 (16)	31 (14)	11 (24)	
Highest level of education				
High school	85 (33)	77 (36)	8 (18)	
Technical college	68 (26)	59 (28)	9 (20)	
University	106 (41)	78 (36)	28 (62)	
Age at menarche, years (Mean, SD)	13 (2)	13 (2)	13 (2)	
Hysterectomy prior to diagnosis				
No	239 (93)	197 (92)	42 (95)	
Yes	19 (7)	17 (8)	2 (5)	
Charlson Comorbidity Score				
0	214 (83)	176 (82)	38 (84)	
1+	45 (17)	38 (18)	7 (16)	
Medical history				
Coronary heart disease	3 (1)	3 (1)	0	
Hypertension	33 (13)	32 (15)	1 (2)	
Diabetes	10 (4)	10 (5)	0	
Family history of breast or ovarian cancer				
No	205 (80)	167 (79)	38 (84)	
Yes	51 (20)	44 (21)	7 (16)	
MHT use prior to diagnosis				
No	237 (92)	200 (93)	37 (82)	
Yes	22 (8)	14 (7)	8 (18)	

Abbreviations: BMI, body mass index; HGSC, high‐grade serous carcinoma; MHT, menopausal hormone therapy; SD, standard deviation.

^a^ Continuous variables presented as median (interquartile range) and categorical variables presented as *n* (%). Numbers may not add up to total due to missing data.

The relationship between use of MHT after diagnosis and OVS is shown in Table 4. In the time‐varying analysis, MHT use was not significantly associated with an improvement in OVS (HR 0.73, 95% CI 0.41–1.31); results were almost identical for OS (data not shown) and similar for PFS (Supplementary Table 6). In the fixed‐variable analysis, the estimate for MHT use during the first 12 months was 0.89 (95% CI 0.49–1.62) overall, and did not differ appreciably for continuing and new users (compared to never users). Among women with HGSC (*n* = 143), MHT use after diagnosis was not associated with survival (Table 4). We had limited power to evaluate the other histotypes but, among the small group of women with endometrioid and low‐grade serous carcinomas (*n* = 59) the estimate for MHT use during the first 12 months was 1.29 (95% CI 0.18–8.98), compared to 0.11 (95% CI 0.01–1.14) for the other histotypes. All key findings were also seen in the propensity score analysis. Results overall and among women with HGSC were similar when we restricted the analysis to women with a complete or partial response to primary treatment (Supplementary Table 7) and when we excluded women with contraindications to MHT (data not shown).

**TABLE 4:** Use of MHT after diagnosis and ovarian cancer‐specific survival among women ≤55 years at diagnosis (*n* = 259).

			All (*n* = 259)	HGSC (*n* = 143)	LGSC & END (*n* = 59)	Other histotypes (*n* = 57)
MHT use	Total (*n*, %)	Deaths (*n*, %)	HR (95%CI)	HR (95%CI)	HR (95%CI)	HR (95%CI)
MHT use time‐varying						
No	NA^b^	NA^b^	1.0	1.0	1.0	1.0
Yes	NA^b^	NA^b^	0.73 (0.41–1.31)	0.92 (0.46–1.83)	1.28 (0.22–7.27)	0.04 (0.004–0.30)
MHT use^a^						
No	214 (83)	85 (86)	1.0	1.0	1.0	1.0
Yes	45 (17)	14 (14)	0.89 (0.49–1.62)	1.04 (0.51–2.14)	1.29 (0.18–8.98)	0.11 (0.01–1.14)
MHT change^a^						
Never	200 (77)	81 (82)	1.0	1.0	1.0	1.0
Use prior to diagnosis only	14 (5)	4 (4)	0.64 (0.22–1.84)	0.31 (0.07–1.33)	NA	1.40 (0.15–13.5)
Continuous use	8 (3)	4 (4)	0.76 (0.27–2.12)	0.71 (0.25–2.01)	NA	NA
New use	37 (14)	10 (10)	0.84 (0.43–1.64)	0.84 (0.35–2.01)	1.44 (0.21–9.78)	0.11 (0.01–1.16)

*Note*: Models adjusted for age (continuous), body mass index (<25, 25–29, 30+ kg/m^2^), smoking (never, former, current), FIGO Stage (I & II, III & IV), residual disease (none, any) and further adjusted for pre‐diagnosis MHT use (yes, no) except for the analysis of MHT change.

Abbreviations: CI, confidence interval; HR, hazard ratio; MHT: menopausal hormone therapy.

^a^ MHT use during the first year after diagnosis.

^b^ Time‐varying.

^c^ The models did not converge due to an insufficient number of samples.

Among the post‐diagnosis cohort who used MHT after diagnosis, only 21 (47%) completed at least one HRQOL questionnaire both before and after initiating MHT; and 186 (87%) of non‐users completed at least two HRQOL questionnaires after treatment. Women who went on to use MHT reported poorer overall QOL (FACT‐G), higher levels of fatigue (FACIT‐fatigue) and lower sexual satisfaction before they initiated MHT compared to non‐initiators; this difference persisted 1–3 months after initiating MHT (Table 5). Only 12 women completed more than one questionnaire after they initiated MHT, their overall QOL (FACT‐G), FACIT‐fatigue scores and sexual satisfaction remained below those of non‐users, although the differences were not statistically significant. The ISI scores did not differ between users and non‐users. Results were similar when we restricted to women (*n* = 12 users/168 non‐users) who completed all three HRQOL questionnaires (Supplementary Table 8).

**TABLE 5:** Health‐related quality of life among women aged ≤55 who did and did not initiate MHT use after treatment for ovarian cancer.

	Time 0—before MHT initiation^b^			Time 1—1–3 months after MHT initiation^b^			Time 2—subsequent questionnaire^b^		
	Non‐users (*n* = 186)	MHT initiators (*n* = 21)		Non‐users (*n* = 186)	MHT use (*n* = 21)		Non‐users (*n* = 168)	MHT use (*n* = 12)	
Health‐related quality of life measurement^a^	Median (IQR)	Median (IQR)	*P* ^c^	Median (IQR)	Median (IQR)	*P* ^c^	Median (IQR)	Median (IQR)	*P* ^c^
Wellbeing (FACT‐G)	86 (75–98)	73 (67–79)	**0.02**	90 (79–98)	78 (67–88)	**0.01**	88 (72–99)	75 (63–93)	0.1
Functional (max 28)	20 (16–25)	16 (13–21)	0.05	23 (18–26)	18 (14–21)	**0.02**	22 (17–26)	18 (14–22)	0.1
Emotional (max 24)	20 (18–23)	20 (16–21)	0.3	20 (18–22)	18 (16–20)	**0.03**	20 (16–22)	19 (16–21)	0.3
Social/family (max 28)	23 (19–26)	20 (19–25)	0.1	23 (19–27)	21 (18–24)	0.2	23 (18–26)	20 (18–25)	0.5
Physical (max 28)	24 (20–26)	19 (16–23)	**0.01**	25 (21–27)	21 (16–24)	**0.002**	24 (21–27)	23 (17–26)	0.2
Fatigue (max 52)	40 (31–46)	31 (22–38)	**0.004**	43 (35–49)	31 (22–42)	**0.001**	42 (32–48)	28 (25–45)	0.1
Insomnia (ISI, max 28)	5 (0–11)	8 (5–13)	0.1	5 (0–11)	10 (0–11)	0.2	1 (0–11)	10 (0–13)	0.5
Insomnia^d^	*N* (%)	*N* (%)		*N* (%)	*N* (%)		*N* (%)	*N* (%)	
No insomnia	117 (63)	7 (41)	0.1	118 (64)	7 (41)	0.1	96 (60)	3 (37)	0.3
Subclinical/clinical	68 (36)	10 (59)		67 (36)	10 (59)		65 (40)	5 (63)	
FACT‐GS7^e^			**0.03**			**0.02**			0.8
Not satisfied	60 (43)	13 (68)		56 (40)	12 (71)		62 (49)	6 (54)	
Somewhat & satisfied	81 (57)	6 (32)		84 (60)	5 (29)		65 (51)	5 (45)	

*Note*: Bold values denote statistical significance at the *p* < 0.05 level.

Abbreviations: FACIT‐fatigue: Functional Assessment of Chronic Illness Therapy—fatigue (range 0–52); FACT, Functional Assessment of Cancer Therapy; IQR, inter‐quartile range; ISI: Insomnia Severity Index. MHT: menopausal hormone therapy.

^a^ The counts in sub‐categories may not add up to the total number due to missing data. The FACT‐G includes 27 items in 4 sub‐scales providing a total QOL score (0–108), higher scores indicate better QOL. Fatigue was measured using FACIT‐fatigue with higher scores indicating less fatigue.

^b^ Time‐0 was the first questionnaire after treatment; this had to be before starting MHT for users. Time‐1 was the second questionnaire after treatment (never users) or the first after starting MHT (users). Time‐2 was the questionnaire after Time 1.

^c^ Kruskal–Wallis non‐parametric test, Pearson chi‐square test or Fisher exact test as appropriate.

^d^ ISI scores 8–14 (considered mild insomnia) and 15+ (moderate to severe insomnia) were combined as sub‐clinical/clinical insomnia.

^e^ The question regarding sex life: ‘I am satisfied with my sex life’; ‘not at all’ and ‘a little bit’ were combined as ‘not satisfied’; ‘somewhat, quite a bit and very much’ were combined as ‘somewhat/satisfied’.

## 4 DISCUSSION

In this population‐based prospective study of Australian women, pre‐diagnosis MHT use was associated with a 26% improvement in ovarian cancer‐specific survival among peri‐/postmenopausal women. The association was slightly stronger for HGSC and would translate into an average 3.7‐month survival advantage for MHT users versus non‐users at 5 years. The association did not differ by recency or duration of use. While the survival benefit with E‐P/P (HR = 0.67) appeared to be stronger than for E‐only (HR = 0.84), the difference was not statistically significant. Among women with advanced stage HGSC, pre‐diagnosis MHT use was associated with 31% better survival and about 11% of this improvement was mediated through differences in residual disease. Among women who were pre−/perimenopausal or aged ≤55 years at diagnosis with HGSC, MHT use after diagnosis was not associated with a difference in survival; in contrast, the estimates were above 1.0 for endometrioid and low‐grade serous carcinomas. Compared to non‐users, MHT users experienced poorer overall QOL, higher levels of fatigue and lower sexual satisfaction prior to initiating MHT and these differences persisted 1–3 months after starting MHT.

Although it is well established that MHT use is associated with an increased risk of developing ovarian cancer, the role of MHT use in ovarian cancer survival remains uncertain. Our findings are consistent with results from previous studies that observed a 10%–20% non‐significant survival benefit with ever‐use of MHT of any type pre‐diagnosis.^8^, ^9^, ^10^, ^37^ We did not observe any difference between MHT type or recency of use, which is in line with the few studies that have investigated this.^6^, ^10^, ^11^, ^13^, ^37^ Previous studies, including the largest study conducted by the Ovarian Cancer Association Consortium (OCAC), found better survival only among women who used MHT for more than 5 years^10^, ^11^, ^12^; however, in our study, the use of MHT was associated with a significantly better survival, regardless of the duration of use. Possible explanations for this difference include the publication of the Women's Health Initiative (WHI) Study results in 2002 which led to substantial changes in patterns of MHT use,^38^ and the higher proportion of older women and those with HGSC in the OPAL cohort.

The biological mechanism underlying the association between pre‐diagnosis MHT use and ovarian cancer survival remains unclear, but MHT use might be associated with the development of less aggressive tumours and, subsequently, better survival outcomes. The OCAC study (which included the OPAL cohort) showed that about 17% of the survival benefit associated with MHT use among women with advanced stage HGSC could be due to the higher proportion of MHT users with no residual disease after primary cytoreduction^12^; this is comparable to the estimate of 11% in our study. A previous study also noted a decreased risk in mortality with pre‐diagnosis MHT use, but only among those who had optimal tumour debulking.^39^ Another possible explanation might be related to expression of oestrogen receptors (ER) and progesterone receptors (PR) which mediate the effects of oestrogen and progesterone on ovarian cancer cell proliferation and apoptosis.^40^ Notably, a study identified an increased risk of developing ERα + ovarian cancer associated with MHT use.^41^ Additionally, a study using data from the international Ovarian Tumour Tissue Analysis consortium showed that the expression of PR and ER are associated with significantly better survival for women with endometrioid and HGSC cancers.^42^ Further studies are needed to improve our understanding of hormone signalling pathways across different histotypes of ovarian cancer.

Only a small number of studies have examined whether MHT use after diagnosis affects survival among women with ovarian cancer.^9^, ^14^, ^15^, ^16^, ^17^ Of three small RCTs, two found that MHT use was not associated with disease‐free interval or survival.^19^, ^21^ In contrast, the largest RCT with longest follow‐up showed significant improvements in both overall survival (HR = 0.63) and relapse‐free survival (HR = 0.67) for the treated group compared to the control group.^18^ However, only 26 of the treated group (35%) continued using MHT and the median duration of MHT use was 1 year, possibly because about 80% of women in this study were postmenopausal at diagnosis. Furthermore, the survival difference was largely driven by the difference between non‐ovarian cancer deaths in the treated and control groups.

The estimates for the association between MHT use after diagnosis and survival among women younger than 55 years in our study are in line with previous observational studies of similar age groups. The only observational study that examined MHT use both before and after diagnosis of ovarian cancer reported that MHT use after diagnosis was associated with a significant 43% lower mortality, but the authors could not rule out selection bias.^9^ They also observed significantly better survival for women with serous and other tumours but not mucinous or endometrioid tumours. In contrast, we did not observe any association between post‐diagnosis MHT use and survival among women with HGSC. Our findings of non‐significant increased mortality with MHT use among women with endometrioid and low‐grade serous carcinomas should be interpreted with caution due to limited statistical power.

The primary goal of using MHT is to alleviate menopausal symptoms. Studies have shown that MHT improves menopause‐specific‐QOL, mainly through relief of symptoms in the general population.^43^, ^44^, ^45^, ^46^ However, the role of MHT in HRQOL for women who have or are at risk for ovarian or breast cancer is unclear. Only one study (*n* = 75) assessed MHT use and HRQOL at 6–12 months after ovarian cancer treatment and reported better QOL among MHT users compared to non‐users.^21^ This study however did not provide data on QOL before women started MHT. Due to the limited sample size, we were unable to draw any clear conclusion as to whether systemic MHT improves HRQOL for younger women treated for ovarian cancer. We found that MHT users experienced poorer overall HRQOL, higher levels of fatigue and lower sexual satisfaction prior to and 1–3 months after they initiated MHT compared to non‐users. However, we were unable to assess menopausal symptoms directly and it may be that their menopausal symptoms did improve but this did not have a substantial impact on overall HRQOL, particularly with potential ongoing cancer treatment‐related side effects.

There are several important strengths to our study. First, we have a long duration of follow‐up and we used prospectively collected data on medication use after diagnosis and considered both self‐reported medication use and claims data, thereby reducing recall error. Second, our post‐diagnosis analyses used longitudinal data on MHT use both before and after ovarian cancer diagnosis, which allowed us to control for MHT use prior to diagnosis. Additionally, we avoided immortal time bias by using a time‐varying analysis and by moving the start of follow‐up to 1 year after diagnosis excluding women who died prior to that time. Finally, to our knowledge, this is the first cohort study to report HRQOL both before and after women started MHT after treatment for ovarian cancer.

Limitations include the comparatively limited statistical power especially for analyses of use after diagnosis, HRQOL and subgroup analysis for different histotypes. We have missing data on MHT type in the pre‐diagnosis use analysis; however, there were no notable differences in results based on complete data analysis and analysis using imputed data. Although we collected comprehensive epidemiological data and were able to adjust for a number of confounders, we cannot exclude the effect of residual confounding from unobserved confounders. However, the use of propensity‐scores to minimise the differences between the treated and untreated groups did not alter the overall conclusions. Finally, menopause‐specific HRQOL data were unavailable; but the questionnaires we used in this study are widely used to measure cancer‐related QOL.

## 5 CONCLUSIONS

In conclusion, our study suggests that pre‐diagnosis MHT use is associated with improved survival, particularly among women with HGSC. Differences in residual disease following cytoreductive surgery explained only a small part of this. Among women ≤55 years, the use of MHT following treatment is not associated with poorer survival for women with HGSC. The findings of differences in the relationship by histotype warrant further evaluation. Well‐designed large‐scale observational studies that address immortal time bias and bias due to confounding by indication, or ideally clinical trials are needed to identify the group of women in whom the benefits outweigh the risks. Such investigations are vital to guide clinical decision‐making and optimise the care of women with ovarian cancer.

## AUTHOR CONTRIBUTIONS

**Renhua Na:** Conceptualization; data curation; formal analysis; investigation; methodology; project administration; software; visualization; writing – original draft; writing – review and editing. **Susan J. Jordan:** Conceptualization; data curation; funding acquisition; investigation; methodology; resources; supervision; validation; writing – original draft; writing – review and editing. **Anna DeFazio:** Conceptualization; funding acquisition; investigation; methodology; resources; validation; writing – review and editing. **Merran Williams:** Investigation; resources; writing – review and editing. **Karen Livingstone:** Investigation; resources; writing – review and editing. **Andreas Obermair:** Conceptualization; funding acquisition; investigation; methodology; writing – review and editing. **Michael Friedlander:** Conceptualization; funding acquisition; investigation; resources; writing – review and editing. **Peter Grant:** Conceptualization; funding acquisition; investigation; writing – review and editing. **Penelope M. Webb:** Conceptualization; data curation; formal analysis; funding acquisition; investigation; methodology; project administration; resources; software; supervision; validation; visualization; writing – original draft; writing – review and editing.

## FUNDING INFORMATION

The OPAL study was funded by the National Health and Medical Research Council (NHMRC) of Australia (GNT1025142, GNT1120431), RN was supported by an NHMRC Program Grant (GNT1073898), PM Webb was supported by an NHMRC Fellowship (GNT1173346). The study sponsor had no role in the study design, in the collection, analysis or interpretation of the data or the writing of the manuscript.

## CONFLICT OF INTEREST STATEMENT

PM Webb received funding from AstraZeneca for an unrelated study of ovarian cancer. PM Webb has received a speaker's fee from AstraZeneca (Nov 2021). A DeFazio received an honorarium from AstraZeneca, and research support from AstraZeneca and lllumina, unrelated to the work described in this manuscript. ML Friedlander declares consulting/advisory boards AstraZeneca, Novartis, GSK, MSD; honoraria: AstraZeneca, GSK, MSD, Limbic; research grants to institution: AstraZeneca, Novartis and Beigene. All other authors declare that they have no conflict of interest that are relevant to the content of this article.

## ETHICS STATEMENT

This study received ethics approval from the QIMR Berghofer Human Research Ethics Committee and all relevant treatment centres. Written informed consent was obtained from all participants involved in this study.

## Supporting information


**DATA S1.** Supporting Information.

## Data Availability

The data supporting the findings of this study may be obtained upon reasonable request from the corresponding author.
