# Supplementary material for: Use of menopausal hormone therapy before and after diagnosis and ovarian cancer survival—A prospective cohort study in Australia
Source: Int J Cancer. 2024 Sep 2;156(2):280–92. doi: 10.1002/ijc.35154 (PMC11578076; doi:10.1002/ijc.35154)
Supplement: Supplementary file 1 — DATA S1. Supporting Information. [file IJC-156-280-s001.pdf]

**Supplemental Appendix**

**TITLE: Use of menopausal hormone therapy before and after diagnosis and ovarian cancer survival – a prospective cohort study in Australia**

**AUTHORS**: Renhua Na, Susan J. Jordan, Anna DeFazio, Merran Williams, Karen Livingstone, Andreas Obermair, Michael Friedlander, Peter Grant, Penelope M Webb for the OPAL study group.

## Contents

Supplementary Methods ........ 1

Supplementary Table 1. Characteristics of women with and without missing data for MHT use prior to diagnosis among peri-/postmenopausal women. ........ 2

Supplementary Table 2. MHT use prior to diagnosis and ovarian cancer-specific survival among peri-/post-menopausal women – multiple imputation (n = 690). ........ 3

Supplementary Table 3. MHT use before diagnosis and progression-free survival among peri-/postmenopausal women ........ 4

Supplementary Table 4. MHT use before diagnosis and ovarian cancer specific survival among peri-/postmenopausal women stratified by stage ........ 5

Supplementary Table 5. Total, direct, and indirect effects (mediated by residual disease) of ever use of MHT before diagnosis on ovarian cancer specific survival among women with advanced high-grade serous carcinoma (n = 457) ........ 6

Supplementary Table 6. Use of MHT after diagnosis and progression-free survival among women ≤ 55 years at diagnosis (n = 229)[1]. ........ 7

Supplementary Table 7. Use of MHT after diagnosis and ovarian cancer-specific survival among women ≤55 years with complete or partial response (n = 245). ........ 8

Supplementary Table 8. MHT use after treatment for ovarian cancer among women ≤55 years and quality of life measurements among women with data at all 3 time-points. ........ 9

Supplementary Figure 1. Absolute standardized differences for baseline covariates comparing pre-diagnosis MHT use vs no use in the original and the propensity score weighted sample. ........ 11

Supplementary Figure 2. Absolute standardized differences for covariates comparing MHT use vs no use during the first 12 months after diagnosis in the original and the propensity score weighted sample. ........ 12

References ........ 13

## Supplementary Methods

### Statistical analysis

***Multiple imputation***

Under the assumption of missing at random, we conducted multiple imputation by chained equations with 20 iterations using the MICE package in R. The variables we used in the imputation were confounders, particularly age and hysterectomy, which were strong predictors of missing data (Supplementary Table 1). We also included auxiliary variables including age at last birth, oral contraceptive use, comorbidities and associated medication use (non-steroidal anti-inflammatory medications), family history of ovarian cancer, stage, histotypes and residual disease, as well as the cumulative baseline cause-specific hazard and binary indicator variables for the outcome.[5,6] We imputed 20 datasets and the resulting estimates and standard errors were combined using Rubin's rules.[7]

***Propensity scores***

We applied propensity score (PS) approach to reduce or minimise the effects of confounding by indication and by histotype.[8] A propensity score is defined as the probability of an individual receiving one of the treatment categories of interest conditional on measured pre-treatment covariates. In this study, we applied inverse probability of treatment weighting (IPTW) approach using PS, focusing on the average treatment effect which denotes the average effect of MHT use among all women when every woman is moved from control to treated group. Specifically, we first used generalised boosted modelling (GBM) logistic regression models taking into account of interactions between any variables to calculate PS using TWANG package in R, which was then used to generate weights for both MHT users (1/PS) and non-uses (1/[1 – PS]). We included variables listed in Table 1 or 3 that were related to the outcome [9], except post-diagnosis variables in the pre-diagnosis GBM models. We assessed the distribution of weights among users and non-users and excluded women with extreme value of weights as extremely weights may increase bias and yield estimates with high variance.[10,11] We used standardised effect size to assess the balance of PS among MHT users and non-users; there was good balance across all covariates with <0.15 absolute standardised mean difference not only for covariates included in the GBM models but also stage, histotypes and residual disease for pre-diagnosis analysis. In the post-diagnosis analysis, while the absolute standardised mean difference was >0.25 for some variables (e.g. education, hypertension and diabetes) that were not confounders, additionally adjusting for these variables did not make any appreciable differences. Supplementary Figure 1 and 2 depict the absolute standardised mean difference for each variable before and after IPTW for pre- and post-diagnosis analysis respectively. Finally, we applied doubly robust estimation method by including confounders in the weighted Cox proportional hazard regression model; this method has been demonstrated to perform better as it is robust to misspecification of one (but not both) of PS and regression models.[12,13] We used bootstrapping to calculate 95% CIs using 2000 replications.

**Supplementary Table 1. Characteristics of women with and without missing data for MHT use prior to diagnosis among peri-/postmenopausal women.**

| Variable[1] | Total (n = 690) | Complete data (n = 580) | Missing data[1] (n = 110) |
|---|---|---|---|
| **Age at diagnosis, years** (Mean, SD) | 63 (8) | 63 (8) | 68 (6) |
| | **N (%)** | **N (%)** | **N (%)** |
| **Menopause status** | | | |
| Perimenopause[2] | 44 (6) | 42 (7) | 2 (2) |
| Postmenopause | 646 (94) | 538 (93) | 108 (98) |
| **FIGO Stage** | | | |
| I & II | 168 (24) | 150 (26) | 18 (16) |
| III & IV | 522 (76) | 430 (74) | 92 (84) |
| **Histotype** | | | |
| High-grade serous carcinoma | 499 (77) | 433 (75) | 92 (84) |
| Non high-grade serous carcinoma | 147 (23) | 147 (25) | 18 (16) |
| **Residual disease** | | | |
| None | 384 (55) | 327 (56) | 57 (53) |
| Any | 283 (45) | 253 (44) | 50 (47) |
| **BMI 5 years before diagnosis (kg/m$^2$)** | | | |
| <25 | 287 (42) | 245 (42%) | 42 (38%) |
| 25-29.9 | 234 (34) | 186 (32%) | 48 (44%) |
| 30+ | 169 (24) | 149 (26%) | 20 (18%) |
| **Smoking status 1 year before diagnosis** | | | |
| Never | 363 (53) | 303 (52) | 60 (55) |
| Former | 247 (36) | 202 (35) | 45 (41) |
| Current | 80 (11) | 75 (13) | 5 (5) |
| **Highest level of education** | | | |
| High school | 349 (51) | 283 (49) | 66 (60) |
| Technical college | 171 (25) | 142 (25) | 29 (26) |
| University | 167 (24) | 152 (26) | 15 (14) |
| **Age at menarche, years** (Mean, SD) | 13 (4) | 13 (4) | 13 (2) |
| **Hysterectomy prior to diagnosis** | | | |
| No | 531 (77) | 459 (79) | 72 (68) |
| Yes | 158 (23) | 124 (21) | 34 (32) |
| **Charlson Comorbidity Score** | | | |
| 0 | 505 (73) | 422 (73) | 83 (76) |
| 1 | 119 (17) | 99 (17) | 20 (18) |
| 2+ | 66 (10) | 59 (10) | 7 (6) |
| **Medical history** | | | |
| Coronary heart disease | 22 (3) | 20 (3) | 2 (2) |
| Hypertension | 266 (39) | 215 (37) | 51 (46) |
| Diabetes | 52 (8) | 44 (8) | 8 (7) |
| **Family history of breast or ovarian cancer** | | | |
| No | 559 (81) | 471 (82) | 878 (81) |
| Yes | 127 (19) | 106 (18) | 21 (19) |

[1] The type of MHT was unknown for 104 women (15%), including six women also missing duration of use, and one missing data on the recency of use. An additional six women were missing other variables: MHT duration (n=2), residual disease (n=3), and hysterectomy status (n=1).

**Supplementary Table 2. MHT use prior to diagnosis and ovarian cancer-specific survival among peri-/post-menopausal women – multiple imputation (n = 690).**

| Variable[1] | All | | HGSC | | Non-HGSC | |
|---|---|---|---|---|---|---|
| | HR | 95%CI | HR | 95%CI | HR | 95%CI |
| **MHT use** | | | | | | |
| Never | 1.0 | | 1.0 | | 1.0 | |
| Ever | 0.74 | 0.60-0.93 | 0.69 | 0.55-0.88 | 1.35 | 0.70-2.62 |
| **Recency of use** | | | | | | |
| Former | 0.74 | 0.58-0.96 | 0.68 | 0.52-0.89 | 1.41 | 0.68-2.93 |
| Current/recent | 0.76 | 0.55-1.05 | 0.73 | 0.52-1.02 | 1.25 | 0.46-3.40 |
| **MHT type** | | | | | | |
| ET | 0.85 | 0.62-1.18 | 0.82 | 0.57-1.17 | 1.12 | 0.49-2.56 |
| E-P/P | 0.68 | 0.51-0.90 | 0.62 | 0.46-0.85 | 1.75 | 0.64-4.77 |
| **MHT duration** | | | | | | |
| < 5 years | 0.70 | 0.53-0.94 | 0.63 | 0.46-0.87 | 1.34 | 0.61-2.92 |
| 5+ years | 0.78 | 0.60-1.02 | 0.74 | 0.55-0.98 | 1.27 | 0.50-3.21 |

Abbreviations: CI: confidence interval; E-P/P: oestrogen plus progestin or progestogen-only therapy; ET: oestrogen-alone hormone therapy; HGSC: high-grade serous carcinoma; HR: hazard ratio; MHT: menopausal hormone therapy.

[1] Variables with missing data that were imputed include MHT type, duration and recency of MHT use (total n=106), hysterectomy status (n = 1). All models adjusted for age (<55, 55-59, 60-64, 65-69, 70+), body mass index (< 25, 25-29, 30+ kg/m$^2$) smoking (never, former, current), history of hysterectomy prior to diagnosis (yes, no) and stratified by FIGO Stage (I & II, III & IV). Further adjustment for any other variables did not make any difference.

**Supplementary Table 3. MHT use before diagnosis and progression-free survival among peri-/postmenopausal women**

| Variable[1] | All | | HGSC | | Non-HGSC | |
|---|---|---|---|---|---|---|
| | HR | 95% CI | HR | 95% CI | HR | 95% CI |
| **MHT use** | | | | | | |
| Never | 1.0 | | 1.0 | | 1.0 | |
| Ever | 0.76 | 0.62-0.93 | 0.67 | 0.54-0.83 | 1.55 | 0.86-2.80 |
| **Recency of use** | | | | | | |
| Former | 0.73 | 0.58-0.92 | 0.63 | 0.49-0.81 | 1.43 | 0.72-2.84 |
| Current/recent | 0.82 | 0.62-1.08 | 0.74 | 0.55-1.00 | 1.78 | 0.79-4.02 |
| **MHT type** | | | | | | |
| ET | 0.80 | 0.58-1.10 | 0.78 | 0.55-1.11 | 1.28 | 0.55-2.99 |
| E-P/P | 0.77 | 0.58-1.02 | 0.69 | 0.52-0.93 | 1.63 | 0.62-4.26 |
| **MHT duration** | | | | | | |
| < 5years | 0.72 | 0.55-0.93 | 0.62 | 0.47-0.82 | 1.50 | 0.75-3.02 |
| 5+ years | 0.80 | 0.62-1.02 | 0.72 | 0.55-0.93 | 1.57 | 0.68-3.60 |

Abbreviation**:** CI: confidence interval; E-P/P: oestrogen plus progestin or progestogen-only therapy. ET: oestrogen-alone hormone therapy; HGSC: high-grade serous carcinoma; HR: hazard ratio; MHT: menopausal hormone therapy; OS: overall survival; OVS: ovarian cancer-specific survival; PFS: progression-free survival.

[1] All models adjusted for age (<55, 55-59, 60-64, 65-69, 70+), body mass index (< 25, 25-29, 30+ kg/m$^2$) smoking (never, former, current), history of hysterectomy prior to diagnosis (yes, no) and stratified by FIGO Stage (I & II, III & IV). Further adjustment for any other variables did not make any difference.

**Supplementary Table 4. MHT use before diagnosis and ovarian cancer specific survival among peri-/postmenopausal women stratified by stage**

| Variable[1] | Stage I & II | | | | | | | Stage III & IV | | | | | | |
|---|---|---|---|---|---|---|---|---|---|---|---|---|---|---|
| | N (%) | All (n = 168) | | HGSC (N = 66) | | Non-HGSC (n = 102) | | N (%) | All (n = 522) | | HGSC (n = 459) | | Non-HGSC (n = 63) | |
| | | HR | 95% CI | HR | 95% CI | HR | 95% CI | | HR | 95% CI | HR | 95% CI | HR | 95% CI |
| **MHT use** | | | | | | | | | | | | | | |
| Never | 118 (70) | 1.0 | | 1.0 | | 1.0 | | 299 (57) | 1.0 | | 1.0 | | 1.0 | |
| Ever | 50 (30) | 0.46 | 0.16-1.33 | 0.39 | 0.07-2.21 | 0.62 | 0.12-3.13 | 223 (43) | 0.75 | 0.60-0.93 | 0.69 | 0.54-0.87 | 1.61 | 0.74-3.47 |
| **Recency of use** | | | | | | | | | | | | | | |
| Former | 33 (20) | 0.64 | 0.22-1.88 | 0.41 | 0.07-2.49 | 1.47 | 0.26-8.25 | 145 (28) | 0.73 | 0.56-0.95 | 0.67 | 0.51-0.88 | 1.54 | 0.66-3.59 |
| Current/recent | 17 (10) | 0.14 | 0.02-1.26 | 0.33 | 0.02-4.63 | NA[2] | NA[2] | 77 (15) | 0.78 | 0.57-1.08 | 0.74 | 0.53-1.04 | 1.78 | 0.56-5.73 |
| **MHT type** | | | | | | | | | | | | | | |
| ET | 22 (13) | 0.07 | 0.01-0.69 | 0.08 | 0.01-1.58 | NA[2] | NA[2] | 65 (12) | 0.92 | 0.64-1.34 | 0.96 | 0.65-1.43 | 1.37 | 0.41-4.56 |
| E-P/P | 11 (7) | 0.42 | 0.08-2.29 | 0.17 | 0.01-3.59 | 1.69 | 0.06-51.7 | 71 (14) | 0.67 | 0.48-0.93 | 0.62 | 0.44-0.88 | 1.71 | 0.47-6.20 |
| **MHT duration** | | | | | | | | | | | | | | |
| < 5years | 31 (19) | 0.54 | 0.17-1.69 | 0.41 | 0.06-3.00 | 0.60 | 0.11-3.26 | 97 (19) | 0.70 | 0.52-0.95 | 0.63 | 0.46-0.86 | 1.81 | 0.72-4.59 |
| 5+ years | 18 (11) | 0.28 | 0.05-1.67 | 0.30 | 0.02-3.86 | 0.76 | 0.03-17.1 | 119 (23) | 0.78 | 0.59-1.03 | 0.74 | 0.56-0.99 | 1.33 | 0.47-3.74 |

Abbreviation**:** CI: confidence interval; E-P/P: oestrogen plus progestin or progestogen-only therapy. HGSC: high-grade serous carcinoma; ET: oestrogen-alone hormone therapy; HR: hazard ratio; MHT: menopausal hormone therapy.

[1] All models adjusted for age (<55, 55-59, 60-64, 65-69, 70+), body mass index (< 25, 25-29, 30+ kg/$m^2$) smoking (never, former, current), history of hysterectomy prior to diagnosis (yes, no) and stratified by FIGO Stage (I & II, III & IV). Further adjustment for any other variables did not make any difference. Numbers may not add up to total due to missing data.

[2] The models did not converge due to an insufficient number of samples.

**Supplementary Table 5. Total, direct, and indirect effects (mediated by residual disease) of ever use of MHT before diagnosis on ovarian cancer specific survival among women with advanced high-grade serous carcinoma (n = 457)**

| **Effects** | **HR (95% CI)** | **Proportion (%)** |
|---|---|---|
| Total effect | 0.69 (0.55-0.87) | 100 |
| Direct effect | 0.72 (0.60-0.85) | 89 |
| Indirect effect, through residual disease | 0.96 (0.83-1.13) | 11 |

Abbreviation**:** HR: hazard ratio; CI: confidence interval;

[1] All models adjusted for age (<55, 55-59, 60-64, 65-69, 70+), body mass index (< 25, 25-29, 30+ kg/m$^2$) smoking (never, former, current), history of hysterectomy prior to diagnosis (yes, no). Further adjustment for any other variables did not make any difference.

**Supplementary Table 6. Use of MHT after diagnosis and progression-free survival among women ≤55 years at diagnosis (n = 229)[1].**

| MHT use | All (n = 229) HR (95%CI) | HGSC (n = 123) HR (95%CI) | LGSC & END (n=55) HR (95%CI) | Other histotypes (n=51) HR (95%CI) |
|---|---|---|---|---|
| **MHT use time-varying** | | | | |
| No | 1.0 | 1.0 | 1.0 | 1.0 |
| Yes | 0.67 (0.36-1.25) | 0.86 (0.46-1.58) | NA[3] | 0.07 (0.02-0.35) |
| **MHT use during the first 12 months[2]** | | | | |
| No | 1.0 | | 1.0 | 1.0 |
| Yes | 0.78 (0.45-1.35) | 1.29 (0.69-2.43) | 0.32 (0.01-7.29) | 0.03 (0.001-0.80) |
| **MHT change[2]** | | | | |
| Never | 1.0 | 1.0 | 1.0 | 1.0 |
| Use prior to diagnosis only | 0.28 (0.08-0.91) | 0.37 (0.11-1.26) | NA[3] | NA[3] |
| Continuous use | 1.16 (0.46-2.93) | 1.14 (0.45-2.93) | NA[3] | NA[3] |
| New use | 0.58 (0.30-1.10) | 1.00 (0.46-2.18) | 0.32 (0.01-7.29) | 0.03 (0.001-0.80) |

Abbreviations: HR: hazard ratio; CI: confidence internal; HGSC: high-grade serous carcinoma; MHT: menopausal hormone therapy;

Models adjusted for age (continuous), body mass index (<25, 25-29, 30+ kg/$m^2$), smoking (never, former, current), FIGO Stage (I & II, III & IV), residual disease (none, any) and further adjusted for pre-diagnosis MHT use (yes, no) except for the analysis of MHT change.

[1] 29 women progressed within the first 12 months were excluded, further excluded one women with missing data on progression status.

[2] MHT use during the first year after diagnosis.

[3] The models did not converge due to an insufficient number of samples.

**Supplementary Table 7. Use of MHT after diagnosis and ovarian cancer-specific survival among women ≤55 years with complete or partial response (n = 245).**

| **MHT use** | **All (n = 245)** | **HGSC (n = 138)** | **LGSC & END (n=53)** | **Other histotypes (n=54)** |
|---|---|---|---|---|
| | **HR (95%CI)** | **HR (95%CI)** | **HR (95%CI)** | **HR (95%CI)** |
| **MHT use time-varying** | | | | |
| No | 1.0 | 1.0 | 1.0 | 1.0 |
| Yes | 0.65 (0.34-1.25) | 0.85 (0.41-1.77) | 0.98 (0.09-10.3) | NA |
| **MHT use during the first 12 months[1]** | | | | |
| No | 1.0 | 1.0 | 1.0 | 1.0 |
| Yes | 0.86 (0.44-1.67) | 0.99 (0.47-2.10) | 0.90 (0.07-12.1) | 0.24 (0.03-1.87) |
| **MHT change[1]** | | | | |
| Never | 1.0 | 1.0 | 1.0 | 1.0 |
| Use prior to diagnosis only | 0.49 (0.15-1.62) | 0.32 (0.07-1.41) | NA[2] | NA[2] |
| Continuous use | 0.58 (0.18-1.88) | 0.52 (0.16-1.72) | NA[2] | NA[2] |
| New use | 0.80 (0.38-1.69) | 0.88 (0.37-2.12) | 1.05 (0.07-14.8) | NA[2] |

Abbreviations: END: endometrioid carcinoma; CI: confidence internal; HGSC: high-grade serous carcinoma; HR: hazard ratio; MHT: menopausal hormone therapy;

Models adjusted for age (continuous), body mass index (<25, 25-29, 30+ kg/m$^2$), smoking (never, former, current), FIGO Stage (I & II, III & IV), residual disease (none, any) and further adjusted for pre-diagnosis MHT use (yes, no) except for the analysis of MHT change.

[1] MHT use during the first year after diagnosis.

[2] The models did not converge due to an insufficient number of samples.

**Supplementary Table 8. MHT use after treatment for ovarian cancer among women ≤55 years and quality of life measurements among women with data at all 3 time-points.**

| Health-related quality of life measurement1 | Time 0 – before MHT initiation[2] | | *P*[3] | Time 1 – 1-3 months after MHT initiation[2] | | *P*[3] | Time 2 – subsequent questionnaire[2] | | *P*[3] |
|---|---|---|---|---|---|---|---|---|---|
| | **Non-users (n = 168)** | **MHT initiators (n = 12)** | | **Non-users (n = 168)** | **MHT use (n = 12)** | | **Non-users (n = 168)** | **MHT use (n = 12)** | |
| | Median (IQR) | Median (IQR) | | Median (IQR) | Median (IQR) | | Median (IQR) | Median (IQR) | |
| **Wellbeing (FACT-G)** | 85 (73-96) | 73 (67-81) | 0.1 | 89 (79-98) | 77 (67-88) | **0.04** | 88 (72-99) | 75 (63-93) | 0.1 |
| Functional (max 28) | 20 (16-25) | 15 (11-21) | 0.1 | 23 (18-26) | 18 (14-20) | **0.04** | 22 (17-26) | 18 (14-22) | 0.1 |
| Emotional (max 24) | 20 (18-23) | 20 (17-21) | 0.9 | 20 (18-22) | 18 (16-20) | 0.1 | 20 (16-22) | 19 (16-21) | 0.3 |
| Social/family (max 28) | 23 (19-26) | 20 (17-24) | 0.2 | 23 (19-27) | 21 (17-24) | 0.3 | 23 (18-26) | 20 (18-25) | 0.5 |
| Physical (max 28) | 24 (19-26) | 19 (17-24) | 0.1 | 25 (21-27) | 21 (17-24) | **0.02** | 24 (21-27) | 23 (17-26) | 0.2 |
| **Fatigue (max 52)** | 40 (30-46) | 30 (21-38) | 0.05 | 43 (35-48) | 32 (22-41) | **0.01** | 42 (32-48) | 28 (25-45) | 0.1 |
| **Insomnia** (ISI, max 28) | 6 (0-11) | 6 (0-11) | 1.0 | 6 (0-11) | 9 (3-11) | 0.6 | 1 (0-11) | 10 (0-13) | 0.5 |
| **Insomnia**[4] | **N (%)** | **N (%)** | | **N (%)** | **N (%)** | | **N (%)** | **N (%)** | |

| | | | | | | | | | |
|---|---|---|---|---|---|---|---|---|---|
| No insomnia | 98 (61) | 5 (63) | 1.0 | 100 (62) | 3 (37) | 0.3 | 96 (60) | 3 (37) | 0.3 |
| Subthreshold/clinical | 63 (39) | 3 (37) | | 61 (38) | 5 (63) | | 65 (40) | 5 (63) | |
| **FACT-GS7**[5] | | | 0.05 | | | 0.3 | | | 0.8 |
| Not satisfied | 57 (44) | 8 (80) | | 50 (39) | 6 (60) | | 62 (49) | 6 (54) | |
| Somewhat/satisfied | 71 (56) | 2 (20) | | 78 (61) | 4 (40) | | 64 (51) | 5 (45) | |

Abbreviations: FACIT-fatigue: Functional Assessment of Chronic Illness Therapy – fatigue (range 0-52); FACT: Functional Assessment of Cancer Therapy. IQR, inter-quartile range; ISI: Insomnia Severity Index. MHT: menopausal hormone therapy.

[1] The counts in sub-categories may not add up to the total number due to missing data. The FACT-G includes 27 items in 4 sub-scales providing a total quality of life (QOL) score (0-108), higher scores indicate better QOL. Fatigue was measured using FACIT-fatigue with higher scores indicating less fatigue.

[2] Time-0 was the first questionnaire after treatment; this had to be before starting MHT for users. Time-1 was the second questionnaire after treatment (never users) or the first after starting MHT (users). Time-2 was the questionnaire after Time 1.

[3] Kruskal–Wallis non-parametric test, Pearson chi-square test or Fisher exact test as appropriate.

[4] ISI scores 8-14 (considered mild insomnia) and 15+ (moderate to severe insomnia) were combined as sub-clinical/clinical insomnia.

[5] The question regarding sex life: "I am satisfied with my sex life"; "not at all" and "a little bit" were combined as "not satisfied"; "somewhat, quite a bit and very much" were combined as "somewhat/satisfied".

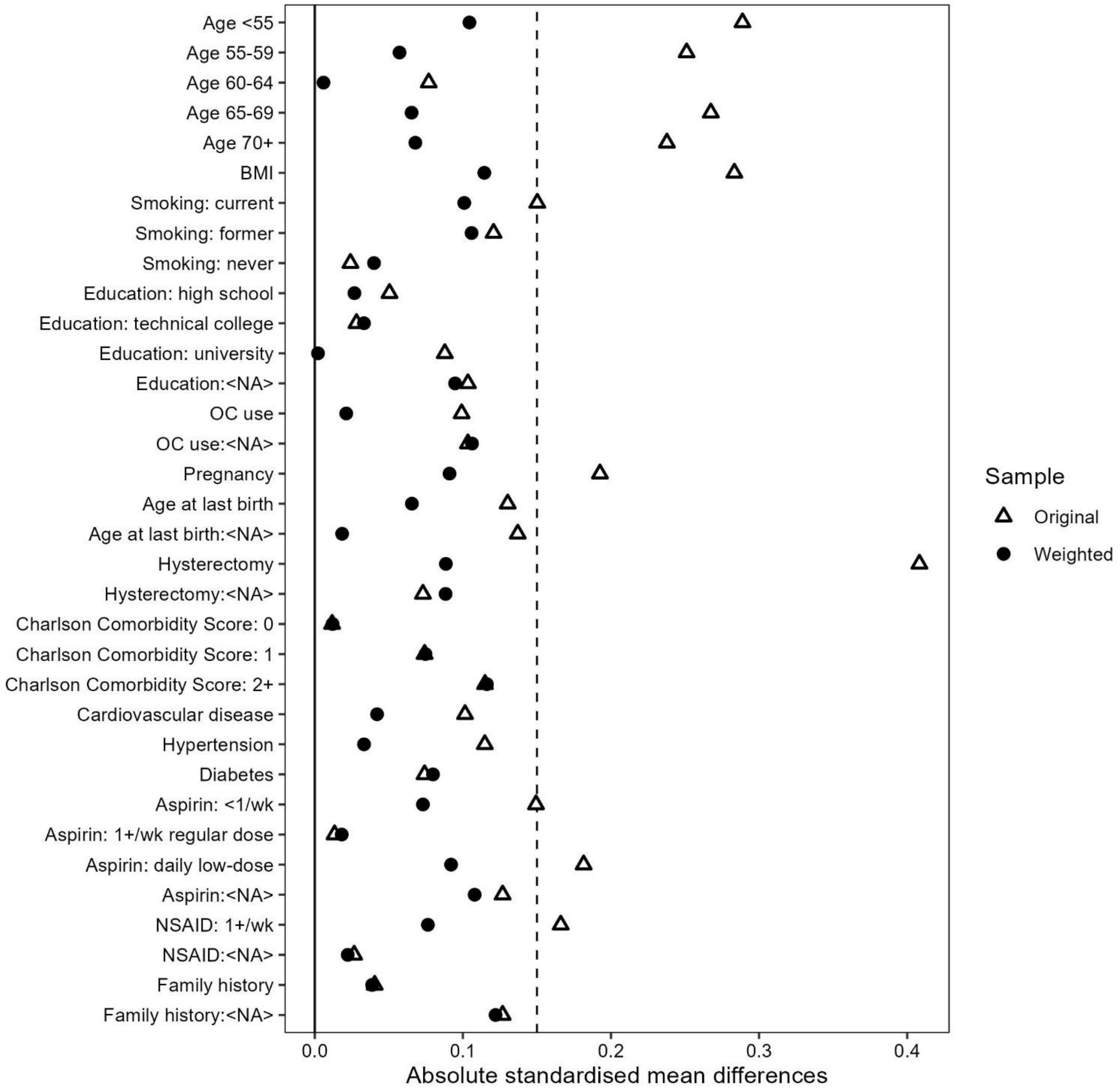


**Supplementary Figure 1. Absolute standardized differences for baseline covariates comparing pre-diagnosis MHT use vs no use in the original and the propensity score weighted sample.**

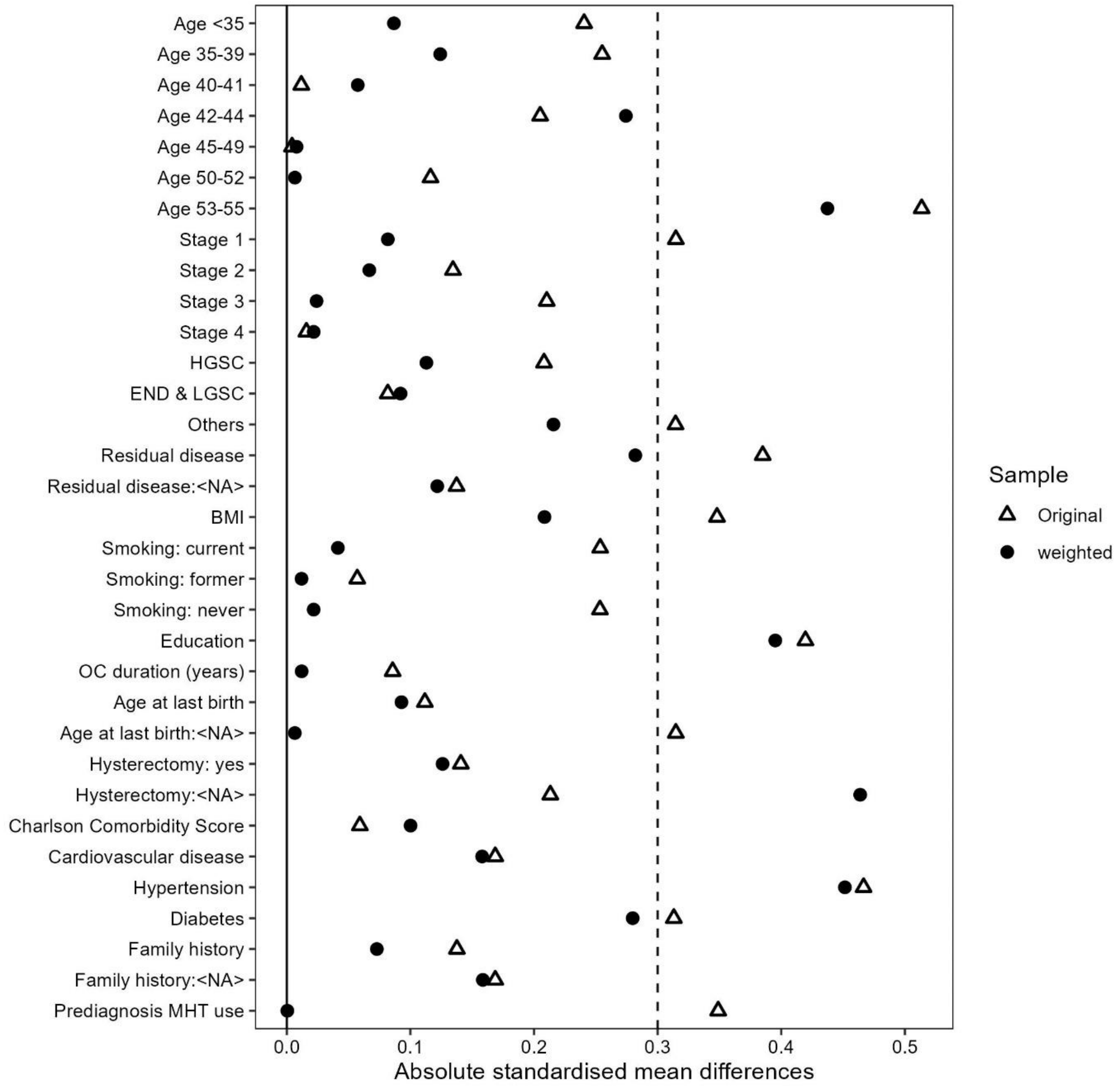


**Supplementary Figure 2. Absolute standardized differences for covariates comparing MHT use vs no use during the first 12 months after diagnosis in the original and the propensity score weighted sample.**

## References

1. Morin CM, Belleville G, Belanger L, et al: The Insomnia Severity Index: psychometric indicators to detect insomnia cases and evaluate treatment response. Sleep 34:601-8, 2011

2. Cella DF, Tulsky DS, Gray G, et al: The Functional Assessment of Cancer Therapy scale: development and validation of the general measure. J Clin Oncol 11:570-9, 1993

3. Yellen SB, Cella DF, Webster K, et al: Measuring fatigue and other anemia-related symptoms with the Functional Assessment of Cancer Therapy (FACT) measurement system. J Pain Symptom Manage 13:63-74, 1997

4. Savard MH, Savard J, Simard S, et al: Empirical validation of the Insomnia Severity Index in cancer patients. Psychooncology 14:429-41, 2005

5. White IR, Royston P: Imputing missing covariate values for the Cox model. Stat Med 28:1982-98, 2009

6. White IR, Royston P, Wood AM: Multiple imputation using chained equations: Issues and guidance for practice. Stat Med 30:377-99, 2011

7. Rubin DB: Multiple imputation for nonresponse in surveys. New York, Wiley, 1987

8. Austin PC, Stuart EA: The performance of inverse probability of treatment weighting and full matching on the propensity score in the presence of model misspecification when estimating the effect of treatment on survival outcomes. Stat Methods Med Res 26:1654-1670, 2017

9. Brookhart MA, Schneeweiss S, Rothman KJ, et al: Variable selection for propensity score models. Am J Epidemiol 163:1149-56, 2006

10. Sturmer T, Rothman KJ, Avorn J, et al: Treatment effects in the presence of unmeasured confounding: dealing with observations in the tails of the propensity score distribution--a simulation study. Am J Epidemiol 172:843-54, 2010

11. Li F, Thomas LE, Li F: Addressing Extreme Propensity Scores via the Overlap Weights. Am J Epidemiol 188:250-257, 2019

12. Linden A: Improving causal inference with a doubly robust estimator that combines propensity score stratification and weighting. J Eval Clin Pract 23:697-702, 2017

13. Lunceford JK, Davidian M: Stratification and weighting via the propensity score in estimation of causal treatment effects: a comparative study. Stat Med 23:2937-60, 2004
